# Decoupling Thermoelectric Performance and Stability in Liquid‐Like Thermoelectric Materials

**DOI:** 10.1002/advs.201901598

**Published:** 2019-10-19

**Authors:** Tao Mao, Pengfei Qiu, Ping Hu, Xiaolong Du, Kunpeng Zhao, Tian‐Ran Wei, Jie Xiao, Xun Shi, Lidong Chen

**Affiliations:** ^1^ State Key Laboratory of High Performance Ceramics and Superfine Microstructure Shanghai Institute of Ceramics Chinese Academy of Sciences Shanghai 200050 China; ^2^ Center of Materials Science and Optoelectronics Engineering University of Chinese Academy of Sciences Beijing 100049 China; ^3^ State Key Laboratory of Metal Matrix Composites School of Materials Science and Engineering Shanghai Jiao Tong University Shanghai 200240 China

**Keywords:** Cu_2_S, liquid‐like materials, service stability, thermoelectric

## Abstract

Liquid‐like materials are one family of promising thermoelectric materials discovered in the past years due to their advantanges of ultrahigh thermoelectric figure of merit (*zT*), low cost, and environmental friendliness. However, their practial applications are greatly limited by the low service stability from the Cu/Ag metal deposition under large current and/or temperature gradient. Both high *zT* for high efficiency and large critical voltage for good stability are required for liquid‐like materials, but they are usually strongly correlated and hard to be tuned individually. Herein, based on the thermodynamic analysis, it is shown that such a correlation can be decoupled through doping immobile ions into the liquid‐like sublattice. Taking Cu_2−_ *_δ_*S as an example, doping immobile Fe ions in Cu_1.90_S scarcely degrades the initial large critical voltage, but significantly enhances the *zT* to 1.5 at 1000 K by tuning the carrier concentration to the optimal range. Combining the low‐cost and environmentally friendly features, these Fe‐doped Cu_2−_ *_δ_*S‐based compounds show great potential in civil applications. This study sheds light on the realization of both good stability and high performance for many other liquid‐like thermoelectric materials that have not been considered for real applications before.

## Graphical abstract

Herein, the correlation between thermoelectric figure of merit and critical voltage is revealed and the thermodynamic analysis suggests that it can be decoupled through doping immobile ions into the liquid‐like sublattice. Along this direction, a new approach to design and fabricate liquid‐like materials with both high thermoelectric performance and good stability is proposed and well demonstrated in Cu_2−_ *_δ_*S‐based liquid‐like materials.

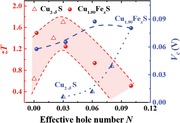

## 1 Introduction

**Figure 1:**
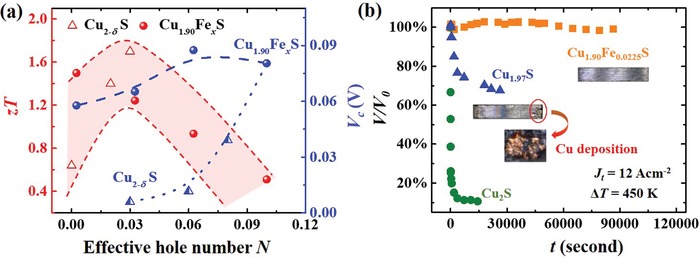
TE performance and service stability. a) TE figure of merit (*zT*) at 1000 K and critical voltage (*V*_c_) under the temperature difference (Δ*T*) of 450 K as a function of the effective hole numbers (*N*) for Cu_2−_ *_δ_*S (δ = 0, 0.03, 0.06, 0.08, and 0.1) and Cu_1.90_Fe*_x_*S (*x* = 0, 0.0125, 0.0225, and 0.0325). *N* = δ for Cu_2−_ *_δ_*S and *N* = δ − 3*x* for Cu_2−_ *_δ_*Fe*_x_*S. b) Relative voltage variations (*V*/*V*_0_) of Cu_1.90_Fe_0.0225_S, Cu_1.97_S, and Cu_2_S as a function of current stress duration (*t*) under *∆T* = 450 K and a current density *J*_t_ = 12 A cm^−2^. The insets show the optical images of Cu_1.90_Fe_0.0225_S and Cu_1.97_S after test. Obvious Cu deposition is observed at the cathode of Cu_1.97_S.

Thermoelectric (TE) technique can realize the direct conversion between heat and electricity, providing an alternative way to more efficiently use fossil energy.^1^, ^2^, ^3^, ^4^, ^5^, ^6^, ^7^, ^8^, ^9^, ^10^, ^11^, ^12^ The energy conversion efficiency of TE technique is dependent on the TE material's dimensionless TE figure of merit *zT* (*zT = S* ^2^ *σT*/κ), where *S* is Seebeck coefficient, σ is electrical conductivity, *T* is absolute temperature, and κ is thermal conductivity. Recently, many Cu‐ and Ag‐based liquid‐like compounds^13^, ^14^, ^15^ (e.g., Cu_2−_ *_δ_X* (*X* = S, Se, Te),^16^, ^17^, ^18^, ^19^ CuAgSe,^20^ Cu_5_FeS_4_,^21^, ^22^ AgCrSe_2_,^23^, ^24^ Cu_7_PSe_6_,^25^ and Ag_9_GaSe_6_ 26, 27) have attracted great attention in TE community due to their extremely low lattice thermal conductivities and high *zT*s. However, the real applications of these liquid‐like compounds are still limited by the overwhelming concerns on their stability and reliability during long‐term service.^28^, ^29^, ^30^, ^31^, ^32^ Under large external field (current and/or temperature gradient), the liquid‐like Cu or Ag ions may deposit on the surface of cathode to form Cu or Ag metal, which will alter material's initial chemical composition and deteriorate its TE performance. In the last century, the 3M Corporation, General Atomics Corporation, Teledyne Energy Systems, and NASA Jet Propulsion Laboratory spent more than 10 years on the Cu_1.97_Ag_0.03_Se_1+_ *_y_*‐based radioisotope thermal generators, but the problem of Cu or Ag ions deposition was never solved. Thus, in 1981, NASA stopped the program of Cu_1.97_Ag_0.03_Se_1+_ *_y_*‐based radioisotope thermal generators.^33^ Until most recently, it is found that the deposition ability of Cu or Ag ions is determined by material's critical voltage (*V*_c_), which represents the threshold when Cu or Ag metal deposition occurs.^34^, ^35^ Higher *V*_c_ corresponds to better stability. Thus, in order to stably use these liquid‐like TE materials in practice application, both high *zT* and large *V*_c_ are required, but they strongly couple in liquid‐like TE materials for given chemical compositions. For example, in Cu_2−_ *_δ_*S (where δ is the Cu off‐stoichiometry, 0 ≤ δ ≤ 0.2), with increasing δ from 0 to 0.2, the *V*_c_ is significantly enhanced but the *zT* is first increased to 1.7 at 1000 K for δ = 0.03 and then quickly decreased to below 1.0 when δ is larger than 0.1 (see **Figure** **1**a). It is impossible to achieve both good *zT* and high *V*_c_ in Cu_2−_ *_δ_*S simultaneously. Although the strategy of introducing electron‐conducting but ion‐blocking interfaces was proposed recently to improve material's stability, such interfaces are hardly fabricated and controlled in bulk materials that are urgently required for TE modules and devices. Therefore, decoupling *zT* and *V*_c_ is the key task for these high‐performance liquid‐like TE materials before any practical application. Herein, the correlation between *zT* and *V*_c_ is revealed based on the thermodynamic analysis and it is found that they can be decoupled through doping immobile ions into the liquid‐like sublattice. Along this direction, a new approach to design and fabricate liquid‐like materials with both high TE performance and good stability is proposed and well demonstrated in Cu_2−_ *_δ_*S‐based liquid‐like TE materials (Figure 1).

## 2 Results and Discussion

When a directional force or field is applied on the Cu‐based liquid‐like material, the mobile Cu ions form a concentration gradient yielding a voltage between the cathode and anode of the material. Cu ions will deposit when the voltage reaches the material's critical voltage *V*_c_. In isothermal case, the *V*_c_ is determined by the Cu off‐stoichiometry δ according to the equation^36^, ^37^

(1) $$V_{c}=\;-\frac{1}{F}\Delta \;\mu_{\mathrm{Cu}}^{\mathrm{crit}}=\;-\frac{RT}{F}\left(\mathrm{Arsinh}\left(\frac{\delta_{c}}{2\;\sqrt{K_{e}}}\right)\;-\;\mathrm{Arsinh}\left(\frac{2\delta -\delta_{c}}{2\;\sqrt{K_{e}}}\right)\right)$$

where $\Delta \mu_{\mathrm{Cu}}^{\mathrm{crit}}$ is critical chemical potential difference, *F* is the Faraday's constant, *K*_e_ is the equilibrium constant for electrons and holes that is independent of stoichiometry, *R* is the gas constant, *T* is the temperature, and δ_c_ is the critical Cu off‐stoichiometry. A larger δ means of a higher *V*_c_ and better stability. Being different with the mobile Cu ions, the immobile ions have little contribution to the voltage under current and/or temperature gradient because they will not form obvious concentration gradient inside the material. However, the immobile ions would act the same role with Cu ions to provide electrons to tune the effective hole number. The above thermodynamic analysis clearly provides a possibility to decouple the *zT* and *V*_c_ in Cu‐based liquid‐like materials: doping immobile ions at the liquid‐like Cu‐sublattice to optimize carrier concentration for high *zT* while maintaining high δ value for large *V*_c_.

Among nunerous Cu‐based liquid‐like compounds, Cu_2−_ *_δ_*S is quite promising for civil applications regarding its unique combination of elements that are low cost, nontoxic, and earth‐abundant. Its maximum *zT* is around 1.7 when δ = 0.03 at 1000 K, superior to those high temperature p‐type TE materials, such as *zT* = 1.5 for FeNb_0.88_Hf_0.12_Sb at 1200 K and *zT* = 1.3 for SiGe at 1200 K.^17^, ^38^, ^39^ When δ is larger than 0.03, the *zT* is quickly reduced due to the increased effective hole number (*N*, where *N* = δ for Cu_2−_ *_δ_*S) (see Figure 1a). However, the *V*_c_ in Cu_2−_ *_δ_*S is monotonously increased when enhancing the δ value by reducing the chemical potential of Cu ions (Figure S1, Supporting Information). Thus, it is impossible to simultaneously achieve both high *zT* and large *V*_c_ in Cu_2−_ *_δ_*S. For example, Cu_1.97_S has a peak *zT* of 1.7 at 1000 K but its *V*_c_ is only 0.006 V when the temperature difference (Δ*T*) between material's two ends is 450 K (hot side temperature *T*_hot_ = 750 K). In contrast, Cu_1.90_S has a large *V*_c_ of 0.08 V when Δ*T* = 450 K, but its *zT* is only 0.5 at 1000 K due to the over‐high effective hole number *N* (see Figure 1a).

**Figure 2:**
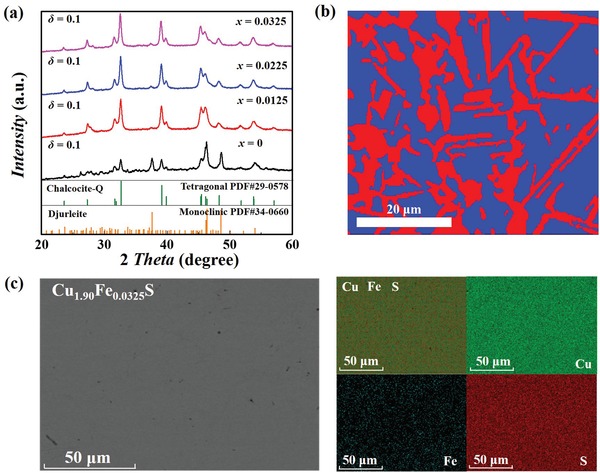
Phase composition and elemental distribution. a) Room‐temperature powder X‐ray diffraction patterns for Cu_2−_ *_δ_*Fe*_x_*S (δ = 0.1, *x* = 0, 0.0125, 0.0225, and 0.0325). b) Phase map of Cu_1.90_Fe_0.0325_S obtained from electron backscatter diffraction (EBSD) measurement. The red and blue grains are identified as djurleite phase and tetragonal chalcocite‐Q phase, respectively. c) Secondary electron (SE) image and elemental energy dispersive spectroscopy (EDS) mapping for Cu_1.90_Fe_0.0325_S.

We dope immobile Fe ions into Cu_2−_ *_δ_*S liquid‐like materials to demonstrate the idea proposed above. Cu_1.90_S is selected as the matrix material because it has a large *V*_c_ (0.08 V when Δ*T* = 450 K) but low *zT* (0.5 at 1000 K). **Figure** **2**a shows the room temperature powder X‐ray diffraction (PXRD) patterns for Cu_1.90_Fe*_x_*S (*x* = 0, 0.0125, 0.0225, and 0.0325). Two different phases are detected, which are identified as the djurleite phase (P21/n) and tetragonal chalcocite‐Q phase (P4_3_2_1_2). This is further confirmed by the electron backscatter diffraction (EBSD) characterization performed on Cu_1.90_Fe_0.0325_S. As shown in Figure 2b, the djurleite phase is randomly distributed inside the tetragonal chalcocite‐Q phase in micrometer scale. The Fe solution contents in these two phases are slightly different, but Cu and S are homogeneously distributed inside each phase (Figure S2 and Table SI, Supporting Information). These results show that Cu_2−_ *_δ_*Fe*_x_*S are polymorph materials consisting of different phases with very close chemical compositions but different crystal structures at room temperature. Similar phenomenon has been also observed in Cu_2_Se_1−_ *_x_*S*_x_* liquid‐like materials.^40^ Likewise, the changed diffraction peak intensity in the PXRD patterns shown in Figure 2a suggests that the proportion of tetragonal chalcocite‐Q phase in Cu_1.90_Fe*_x_*S gradually increases with increasing the Fe‐doping content. The differential scanning calorimetric (DSC) measurements shown in Figure S3 (Supporting Information) suggest that all Cu_2−_ *_δ_*Fe*_x_*S materials finally convert to a cubic antifluorite structure at elevated temperature (above 650 K). This can be confirmed by the high‐temperature PXRD performed on Cu_1.90_Fe_0.0325_S (see Figure S4, Supporting Information). Likewise, these phase transitions are reversible, as confirmed by the well consistency between the exothermic peaks in the cooling process and the endothermic peaks in the heating process shown in Figure S5 (Supporting Information).

**Figure 3:**
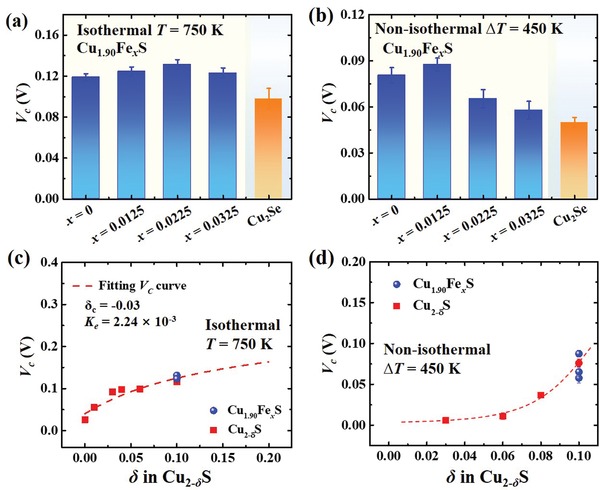
Critical voltage under both isothermal case and nonisothermal case. Experimentally determined critical voltage (*V*_c_) for Cu_1.90_Fe*_x_*S (*x* = 0, 0.0125, 0.0225, and 0.0325) in the a) isothermal case with a constant temperature of 750 K and b) nonisothermal case with a temperature difference *∆T* = 450 K (hot side temperature *T*_hot_ = 750 K). The data for Cu_2_Se are included for comparison. *V*_c_ as a function of Cu off‐stoichiometry δ for Cu_2−_ *_δ_*Fe*_x_*S and Cu_2−_ *_δ_*S in the c) isothermal case with a constant temperature of 750 K and d) nonisothermal case with a temperature difference *∆T* = 450 K (hot side temperature *T*_hot_ = 750 K). The *V*_c_ for Cu_2−_ *_δ_*Fe*_x_*S samples are almost the same and thus the data points in (c) overlap with each other. The dashed line in (c) represents the theoretical *V*_c_ 35 curve predicated by Equation (1) with δ_c_ = −0.03 and *K*_e_ = 2.24 × 10^−3^. The dashed line in (d) is a guide to the eyes.

Via monitoring material's relative electrical resistance variation (*R*/*R*_0_, where *R*_0_ is the material's initial electrical resistance) and relative voltage variation (*V*/*V*_0_, where *V*_0_ is the material's initial voltage) before and after applying different electric currents (*J*) on the sample, *V*_c_ in the isothermal condition and in the nonisothermal condition can be obtained based on the knee point found in the measured *R*/*R_0_* versus *J* curve and *V*/*V*_0_ versus *J* curve, respectively. **Figure** **3**a shows the measured *V*_c_ values in the isothermal condition under a constant temperature of 750 K. As expected, the Fe‐doped Cu_2−_ *_δ_*S samples possess the *V*_c_ values around 0.12 V, which are similar with that for Cu_1.90_S. These values are also comparable to that of Cu_2_Se, which has been successfully used for the fabrication of stable TE module.^41^ Figure 3b shows the measured *V*_c_ values for Cu_1.90_Fe*_x_*S in the nonisothermal condition with the temperature difference *∆T* = 450 K between the two ends of materials (hot side temperature *T*_hot_ = 750 K). All *V*_c_ values are in the range of 0.05–0.08 V, which are also comparable with those of Cu_1.90_S and Cu_2_Se in the same condition. Figure 3c,d shows the *V*_c_ as a function of Cu off‐stoichiometry δ in the isothermal condition and in the nonisothermal condition, respectively. The measured *V*_c_ values quickly increases with increasing δ. In the isothermal condition, the *V*_c_ values for Fe‐doped Cu_2−_ *_δ_*S are almost the same as those of Cu_2−_ *_δ_*S matrix materials with the same δ value. In the nonisothermal condition, the case is similar although the deviation is large due to the contribution of Seebeck effect under large temperature gradient (see the Supporting Information). All these data strongly suggest that the immobile Fe dopants have no or very weak contribution to *V*_c_, although they can raise the energy barrier that Cu ions have to overcome in order to jump from one equivalent site to another from the point of view of kinetics.^31^ This is quite reasonable based on the thermodynaic analysis shown above.

The high *V*_c_ values observed in Cu_1.90_Fe*_x_*S indicate that they possess good stability under large current and/or temperature gradient. In order to confirm this, long‐term current stress test is performed on Cu_1.90_Fe_0.0225_S under both large temperature gradient (*∆T* = 450 K) and high current density (*J*_t_ = 12 A cm^−2^). Figure 1b shows that the *V*/*V*_0_ for Cu_1.90_Fe_0.0225_S is scarcely changed even after 90 000 s test. Likewise, no obvious cracks or Cu metal deposition are observed on the surface of Cu_1.90_Fe_0.0225_S after the test. In fact, till the current density is raised to 24 A cm^−2^, the *V*/*V*_0_ starts to decrease (Figure S6, Supporting Information). In contrast, the *V*/*V*_0_ values for Cu_1.97_S and Cu_2_S are quickly changed in the current stress test. For example, the *V*/*V*_0_ for Cu_1.97_S is quickly reduced to 74% after about 7200 s under the condition of *∆T* = 450 K and *J*_t_ = 12 A cm^−2^. For Cu_2_S, the *V*/*V*_0_ is quickly reduced to only 10% after just 100 s under the same testing condition. Especially, after the test, the crack appears near the cold side of Cu_1.97_S with some red color Cu metal deposition. These results prove that the Cu_1.90_Fe*_x_*S samples possess much better stability than Cu_1.97_S and Cu_2_S.

Recently, a rational design strategy for TE modules based on liquid‐like materials that enables both good stability and high energy conversion efficiency has been proposed.^41^ Guided by this strategy, assuming the present p‐type Cu_1.90_Fe_0.0325_S is coupled with n‐type Yb_0.3_Co_4_Sb_12_ to assemble the TE module with the ratio of cross‐sectional areas of p‐ and n‐legs (*A*_p_ */A*_n_) equal to 4:1, the *V*_c_ for Cu_1.90_Fe_0.0325_S must be higher than 0.037 V for stable operation under the *∆T* of 450 K. The calculation details can be found elsewhere.^41^ As shown in Figure 3b, the measured *V*_c_ of Cu_1.90_Fe_0.0325_S is 0.058 V under the *∆T* of 450 K, well satisfying the requirement. Thus, the Cu_1.90_Fe_0.0325_S/Yb_0.3_Co_4_Sb_12_ TE module is expected to exhibit good stability during real application.

**Figure 4:**
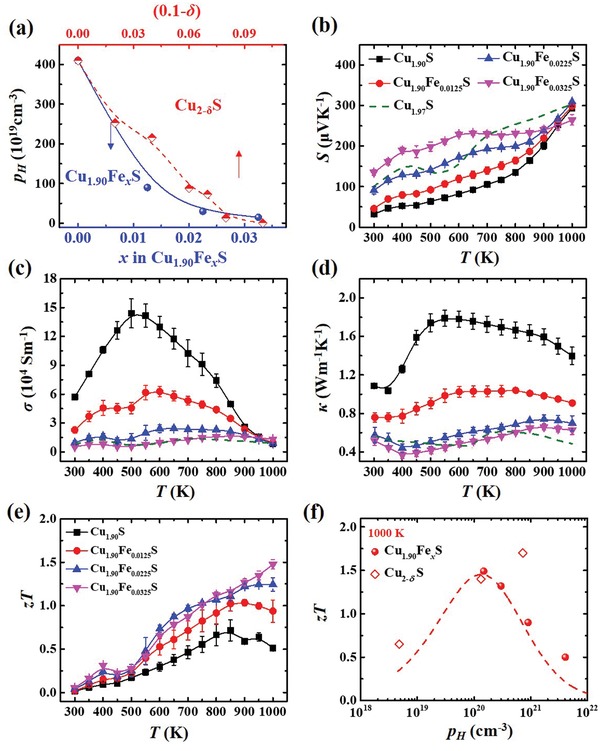
Carrier concentration and TE properties. a) Room‐temperature Hall carrier concentration (*p*_H_) as a function of Fe‐doping content (*x*) in Cu_1.90_Fe*_x_*S (*x* = 0, 0.0125, 0.0225, and 0.0325). The data for Cu_2−_ *_δ_*S (δ = 0, 0.03, 0.06, 0.08, and 0.1) are also included for comparison.^44^ The lines are guides to the eyes. Temperature dependences of b) Seebeck coefficient (*S*), c) electrical conductivity (σ), d) thermal conductivity (κ), and e) TE figure of merit *zT* for Cu_2−_ *_δ_*Fe*_x_*S. f) *p*_H_ dependence of *zT* at 1000 K for Cu_2−_ *_δ_*Fe*_x_*S. The data for Cu_2−_ *_δ_*S are included for comparison. The dashed line represents the theoretical values calculated based on the single parabolic model (SPB). The calculated details can be found in the Supporting Information.

The TE properties can be significantly optimized by doping Fe in Cu_2−_ *_δ_*S. Although Cu_1.90_S has large *V*_c_ and good stability, the large Cu off‐stoichiometry leads to overhigh hole concentration that deviates off the optimal range for high *zT* (Figure 1a). As shown in Figure S7 (Supporting Information), the valence state of Cu in Cu_2−_ *_δ_*S is +1 while it is +3 for Fe.^42^, ^43^ Thus, doping Fe into Cu_1.90_S would introduce additional electrons to reduce the overhigh hole concentration approaching to the optimal range in ideal case. **Figure** **4**a presents that the *p*_H_ for Cu_1.90_S at 300 K is 4.6 × 10^21^ cm^−3^. It is reduced by one order of magnitude to only 1.5 × 10^20^ cm^−3^ for Cu_1.90_Fe_0.0325_S, an optimal carrier concentration for high *zT* in Cu_2−_ *_δ_*S.^17^ Likewise, the carrier mobility is slightly increased with increasing the Fe doping content (Figure S8, Supporting Information).

The reduced hole concentration in Cu_1.90_Fe*_x_*S leads to increased Seebeck coefficient *S* throughout the entire measured temperature range. As shown in Figure 4b, the *S* gradually increases with increasing the Fe‐doping content. The *S* for Cu_1.90_Fe_0.0325_S at 300 K is 147 µV K^−1^, about four times of that for Cu_1.90_S. Likewise, the electrical conductivity σ decreases with increasing the Fe‐doping content. At 300 K, the σ for Cu_1.90_Fe_0.0325_S is 5.1 × 10^3^ Sm^−1^, about one order of magnitude lower than that for Cu_1.90_S. Figure S9 (Supporting Information) shows that the *S* and σ for two batches of Cu_1.90_Fe*_x_*S samples have good consistency. The kinks in the temperature range of 350–550 K are related to the phase transition from the tetragonal chalcocite‐Q phase to cubic phase. The variations of thermoelectric properties induced by phase transitions have been observed in many Cu‐ and Ag‐containing compounds with phase transitions.^45^ Likewise, Figure S10 (Supporting Information) shows that the data and temperature dependence for the *S* and σ of Cu_1.90_Fe_0.0325_S are well reproduced up to 900 K during the cycling test. These results prove that the performances of the present samples are repeatable and reproducible. Based on the measured *S* and σ, the power factors (PF = *S* ^2^σ) for Cu_2−_ *_δ_*Fe*_x_*S are calculated and shown in Figure S11 (Supporting Information). The peak PF for Cu_1.90_Fe_0.0325_S is around 9 µW cm^−1^ K^−2^. This value is comparable with that for Cu_1.97_S,^17^ which has the best *zT* in Cu_2−_ *_δ_*S matrix compounds.

The reduced hole concentration by doping Fe also leads to reduced thermal conductivity κ throughout the entire measured temperature range. As shown in Figure 4d, Cu_1.90_S shows the high κ above 1 Wm^−1^ K^−1^. With increasing the Fe content, κ gradually decreases. The κ for Cu_1.90_Fe_0.0325_S at 300 K is only 0.5 Wm^−1^ K^−1^, about one half of that for Cu_1.90_S. On one hand, such great κ reduction is contributed by the suppressed lattice thermal conductivity (κ_L_) (Figure S12, Supporting Information). Due to the atomic radius and mass difference between Cu and Fe, doping Fe can strengthen the point defect scattering to phonons yielding low κ_L_. On the other hand, the κ reduction is also related with the suppressed carrier thermal conductivity *(κ*_C_) due to the reduced carrier concentration. According to the Wiedemann–Franz law, κ_C_ can be estimated by κ_C_ *= LTσ*, where *L* is the Lorenz number calculated by the single parabolic model.^46^ As shown in Figure S13 (Supporting Information), the calculated κ_C_ significantly decreases with increasing the Fe content throughout the entire measured temperature range due to the reduced σ. For Cu_1.90_S, the proportion of κ_C_/κ at 300 K is 38%. For Cu_1.90_Fe_0.0325_S, it is already reduced to as low as 5%. The role of low carrier concentration on the κ reduction can be more clearly reflected in Figure S14 (Supporting Information). The *κ* for Cu_1.90_Fe_0.0325_S with carrier concentration around 1 × 10^20^ cm^−3^ already reaches the minimum value in Cu_2−_ *_δ_*S‐based materials.

The increased *S* and lowered κ result in greatly enhanced *zT* in Cu_1.90_Fe*_x_*S as compared to Cu_1.90_S. As shown in Figure 4e, the maximum *zT* achieved in Cu_1.90_Fe*_x_*S is 1.5 at 1000 K, comparable with that in Cu_1.97_S and about three times of that in Cu_1.90_S. The great enhancement of *zT* is mainly caused by the optimized carrier concentration, which is clearly illustrated in Figure 4f. More importantly, both high TE performance and good stability are simultaneously achieved in Cu_1.90_Fe*_x_*S (Figure 1a, and Figure S15, Supporting Information). This makes the ternary Cu_1.90_Fe*_x_*S suitable for TE module fabrication.

## 3 Conclusion

In summary, via doping immobile Fe atoms into Cu_1.90_S, we developed a series of Cu_2−_ *_δ_*S‐based liquid‐like materials simultaneously possessing good stability and high *zT*. Combining their low‐cost and environmentally friendly features, these Fe‐doped Cu_2−_ *_δ_*S‐based compounds show great potential in civil applications. Therefore, this study sheds light on the realization of both good stability and high TE performance for many other liquid‐like TE materials that are usually not considered for practical applications before.

## 4 Experimental Section

Polycrystalline Cu_2−_ *_δ_*Fe*_x_*S (δ = 0.1, *x* = 0, 0.0125, 0.0225, and 0.0325) and Cu_2−_ *_δ_*S (δ = 0, 0.01, 0.03, 0.04, 0.06, and 0.1) samples were synthesized by a combination of melting and long‐term high‐temperature annealing method. High purity raw elements, Cu (shot, 99.999%, Alfa Aesar), S (shot, 99.999%, Alfa Aesar), and Fe (shots, 99.98%, Alfa Aesar) were weighed in their stoichiometric ratios and placed in boron nitride crucibles, and then sealed in fused silica tubes under vacuum. The temperature of the tubes was slowly raised to 1423 K in 6 h and then maintained at this temperature for 12 h before quenching into ice water. Then, the ingots were annealed at 773 K for 5 d. The annealed ingots were crushed into powders and consolidated by spark plasma sintering (Sumitomo SPS‐2040) at 723 K under a pressure of 65 MPa for 5 min. Electrically insulating but thermally conducting BN layers were sprayed onto the carbon foils and the inner sides of the graphite die before the SPS process in order to prohibit DC pulsed currents going through the powders.

The phase purity and crystal structure of the fabricated Cu_2−_ *_δ_*Fe*_x_*S (δ = 0.1, *x* = 0, 0.0125, 0.0225, and 0.0325) polycrystalline samples were examined by powder X‐ray diffraction analysis (Rint 2000, Rigaku, Japan) using Cu K_α_ radiation (λ = 1.5405 Å). The measurements were performed between 2θ = 20°–60° with a scan width of 0.02° and a rate of 4° min^−1^. High‐temperature X‐ray diffraction was carried out on a Bruker D8 ADVANCE (Bruker AXS, Germany) from 300 to 750 K. The sample morphology and homogeneity were analyzed by energy dispersive X‐ray analysis (EDS, Oxford Horiba 250). Differential scanning calorimetric measurements (Netzsch DSC 404F3) were performed to illustrate the phase transition characters of the fabricated Cu_2−_ *_δ_*Fe*_x_*S polycrystalline samples. X‐ray photoelectron spectroscopy (XPS, ESCALAB 250, Thermo Scientific) was used to identify the valence state of Fe. X‐rays used in the XPS measurements were produced by a monochromatized Al anode (Al K_α_ = 1486.6 eV). Before XPS measurement, the samples were sputter‐cleaned for 30 s with an Ar^+^ ion beam (4 kV, 10 mA) in vacuum to remove surface contaminants.

The electrical conductivity and Seebeck coefficient were measured by using an Ulvac ZEM‐3 from 300 to 1000 K. The size of the measured sample was about 2 × 2 × 8 mm^3^. The thermal conductivity was calculated from *κ = DC*_p_ρ. The thermal diffusivity (*D*) was obtained by using a laser flash method (Netzsch LFA 457). The thickness and the diameter of the samples were about 1 and 10 mm, respectively. The specific heat (*C*_p_) was estimated by using the Dulong–Petit approximation (*C*_p_ = 3*Nk*_B_) to eliminate the contribution from the phase transitions. The density (ρ) was measured by using the Archimedes method. The uncertainties in the electrical conductivity, Seebeck coefficient, and thermal diffusivity were ±4–9%, ±4%, and ±5–10%, respectively.^47^ However, the repeatability between successive measurements carried out under the same condition was much better when using the commercial instruments.^48^ Hall coefficients (*R*_H_) at room temperature were measured in a Physical Property Measurement System (Quantum Design) by sweeping the magnetic field up to 3 T in both positive and negative field directions. Carrier concentration *p*_H_ was calculated by using *p*_H_ = 1/*eR*_H_, where *e* was the elementary charge. Carrier mobility (*µ*_H_) was calculated according to the relation *µ*_H_ *= σR*_H_. The size of the sample used to be measured in physical property measurement system was about 1 × 2 × 8 mm^3^.

Critical voltage *V*_c_ in isothermal case was measured in a reformed Netzsch DIL 402C equipment by monitoring the material's relative electrical resistance variation (*R*/*R*_0_, where *R*_0_ is the material's initial electrical resistance) before and after applying different electric currents on the sample. The size of the measured sample was 1.5 × 1.5 × 6 mm^3^. The schematic of the measurement is shown in Figure S16a (Supporting Information). The knee point found in the *R*/*R*_0_ versus *J* curve corresponds to the threshold when the Cu metal deposition occurs. The current density at this knee point is the critical current density (*J*_c_). All the stability tests in isothermal case were performed at 750 K when all the Cu_1.90_Fe*_x_*S samples convert into the superionic cubic phase. Figure S16b (Supporting Information) shows the measurement results for the Cu_2−_ *_δ_*Fe*_x_*S samples in the isothermal case with a constant temperature of 750 K. Based on the measured *J*_c_, *V*_c_ can be obtained by the relation *V*_c_ *= J*_c_ *L/σ*, where *L* and σ are the length and electrical conductivity, respectively.

The critical voltage *V*_c_ under thermal gradient was measured in another home‐built instrument by monitoring the material's relative voltage variation (*V*/*V*_0_, where *V*_0_ is the material's initial voltage) before and after applying different electric currents on the sample. The size of the measured sample was 1.5 × 1.5 × 6 mm^3^. The schematic of the measurement is shown in Figure S17a (Supporting Information). The measurement results are shown in Figure S17b (Supporting Information). Based on the *J*_c_ values at the knee points of the *V*/*V*_0_ versus *J* curves, the *V*_c_ with the temperature difference *∆T* = 450 K (hot side temperature *T*_hot_ = 750 K) is calculated by the relation *V*_c_ *= J*_c_ *L*_eff_/σ_avg_, where *L*_eff_ and σ_avg_ are the length and average electrical conductivity of the material with superionic phase under temperature gradient, respectively. The σ_avg_ data are shown in Tables SII and SIII (Supporting Information). It should be noted that these *V*_c_ values are different from that in the previous isothermal condition. It is the additional potential that must be overcome for Cu deposition beyond the potential that generates from the thermo‐diffusion of charged species. For comparison, the *V*_c_ values for Cu_2−_ *_δ_*S (δ = 0.03, 0.06, and 0.08) are also measured and the data are shown in Figure S18 (Supporting Information). All the measurements were conducted in a chamber filled with high pure Argon.

## Conflict of Interest

The authors declare no conflict of interest.

## Supporting information

Supplementary
