# Supplementary material for: Decoupling Thermoelectric Performance and Stability in Liquid‐Like Thermoelectric Materials
Source: Adv Sci (Weinh). 2019 Oct 19;7(1):1901598. doi: 10.1002/advs.201901598 (PMC6947709; doi:10.1002/advs.201901598)
Supplement: Supplementary file 1 — Supplementary [file ADVS-7-1901598-s001.pdf]

Copyright WILEY-VCH Verlag GmbH & Co. KGaA, 69469 Weinheim, Germany, 2019.

ADVANCED SCIENCE Open Access

Supporting Information

for *Adv. Sci.,* DOI: 10.1002/advs.201901598

Decoupling Thermoelectric Performance and Stability in Liquid-Like Thermoelectric Materials

Tao Mao, Pengfei Qiu, Ping Hu, Xiaolong Du, Kunpeng Zhao, Tian-Ran Wei, Jie Xiao, Xun Shi,* and Lidong Chen*

Supporting Information

Decoupling Thermoelectric Performance and Stability in Liquid-like Thermoelectric Materials

Tao Mao, Pengfei Qiu, Ping Hu, Xiaolong Du, Kunpeng Zhao, Tianran Wei, Dudi Ren, Xun Shi*, Lidong Chen*

Dr. P. Qiu, Mr. T. Mao, Mr. P. Hu, Mr. X. Du, Mrs. J. Xiao. Prof. X. Shi, Prof. L. Chen
State Key Laboratory of High Performance Ceramics and Superfine Microstructure, Shanghai Institute of Ceramics, Chinese Academy of Sciences, Shanghai 200050, China
Mr. T. Mao, Mr. P. Hu, Mr. X. Du
Center of Materials Science and Optoelectronics Engineering, University of Chinese Academy of Sciences, Beijing 100049, China
Dr. K. Zhao, Dr. T. Wei,
State Key Laboratory of Metal Matrix Composites, School of Materials Science and Engineering, Shanghai Jiao Tong University, Shanghai, 200240, China

E-mail: qiupf@mail.sic.ac.cn; xshi@mail.sic.cn;

Critical voltage under temperature difference

In the non-isothermal case, the occurrence of Cu metal deposition needs to overcome the additional potential that results from the thermo-diffusion of charged species. When the current is applied in the same direction as the thermal gradient, the critical voltage V_c under temperature difference ΔT is given by[1]

$$V_c = -\frac{1}{F}\,\Delta\mu_{Cu}^{crit} - S^{*}|\Delta T| \qquad (2),$$

where S^* includes the electronic Seebeck coefficient and ionic Seebeck coefficient. In binary $Cu_{2-\delta}S$, both the electronic Seebeck coefficient and ionic Seebeck coefficient will increase with decreasing δ. Thus, the V_c in the non-isothermal case would be more sensitively dependent on δ than that in the isothermal case.

Calculated *zT* based on the SPB model

On the basis of the Fermi statistics, Seebeck coefficient S can be expressed as[2]:

$$S = \frac{k}{e}(\frac{(\lambda + 2)F_{\lambda+1}(\eta)}{(\lambda + 1)F_{\lambda}(\eta)} - \eta)$$

where k_B is the Boltzmann constant, e is the electron charge, λ is the scattering factor with a value of 0 for acoustic phonon scattering, and η ($=E_F/k_BT$) is the reduced Fermi energy. The Fermi integrals are given by $F_m(\eta) = \int_0^{\infty} \frac{x^m dx}{1+\exp(x-\eta)}$, where x is the reduced carrier energy. The Hall carrier concentration p_H can also be expressed as a function of Fermi integrals:

$$p_H = 4\pi \left(\frac{2m^* k_B T}{h^2}\right)^{\frac{3}{2}} \frac{F_{\frac{1}{2}}(\eta)}{r_H}$$

$$r_H = \frac{3}{4} \frac{F_{\frac{1}{2}}(\eta) F_{-\frac{1}{2}}(\eta)}{{F_0}^2(\eta)}$$

where m^* is the electronic effective mass and r_H is the Hall factor

$$L = (\frac{k_B}{e})^2 \left\{\frac{(\lambda+3)F_{\lambda+2}(\eta)}{(\lambda+1)F_{\lambda}(\eta)} - \left[\frac{(\lambda+2)F_{\lambda+1}(\eta)}{(\lambda+1)F_{\lambda}(\eta)}\right]^2\right\} .$$

In the SPB model based on Fermi-Dirac statistics, the drift mobility for acoustic phonon scattering (μ_{ph}) in the non-degenerate limit can be expressed as

$$\mu_{ph} = \frac{(8\pi)^{1/2} e h^4 \rho {v_l}^2}{3(k_B T)^{\frac{3}{2}} (m^*)^{5/2} \Xi^2}$$

where $\hbar$ is the reduced Planck constant, ρ is the sample density, v_l is the velocity of longitudinal sound waves, m^* is the density-of-states effective mass for a single valley, and Ξ is the deformation potential regarded as a measure of the strength of carrier-phonon interaction.

$$\sigma = p_H e \mu$$

$$\kappa_{total} = \kappa_l + L\sigma T$$

$$zT = \frac{S^2 \sigma T}{\kappa_{total}}$$

The κ_L is set as 0.39 $Wm^{-1}K^{-1}$ and the potential deformation Ξ is set as 1.3 eV.

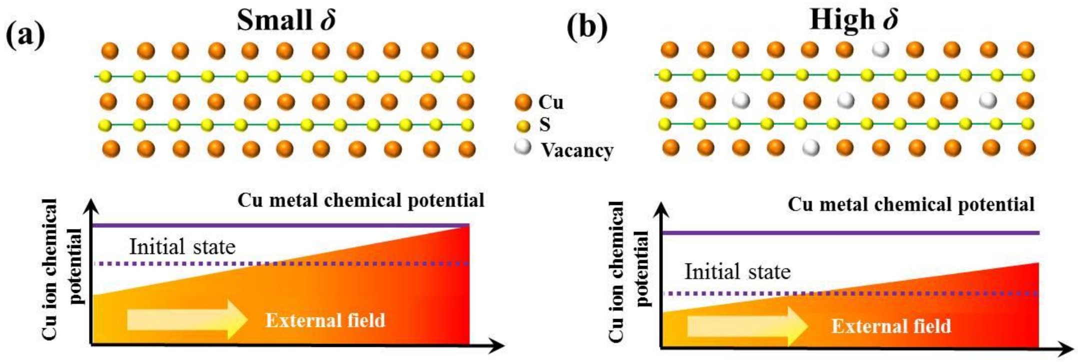

Figure S1 Schematic of the chemical potential of Cu ions under external field for $Cu_{2-\delta}S$ with (a) small Cu off-stoichiometry δ and (b) high Cu off-stoichiometry δ. The higher δ indicates that the chemical potential of Cu ions is more difficult to be raised to that of Cu metal for Cu metal deposition.

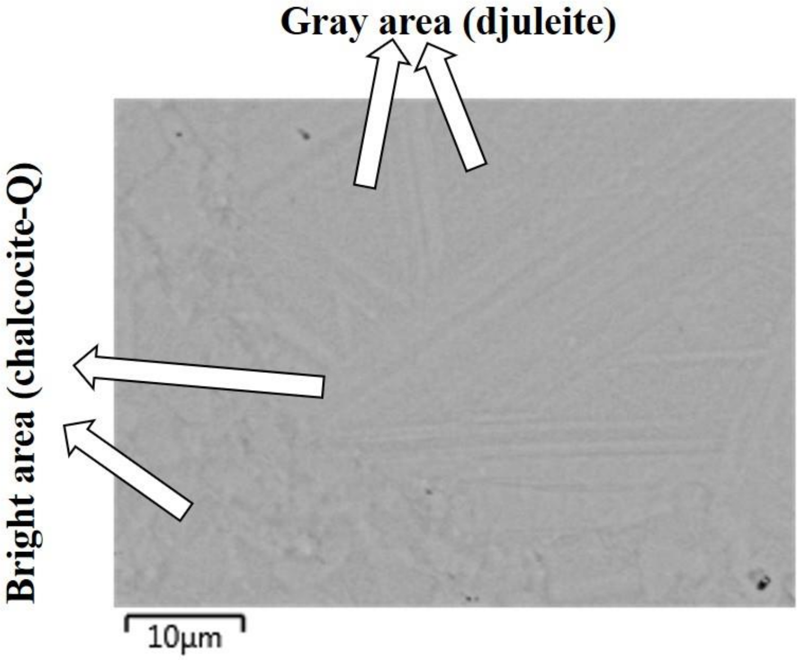

Figure S2 Elemental energy dispersive spectroscopy (EDS) mapping for $Cu_{1.90}Fe_{0.0325}S$. Two slightly different contrasts can be observed, corresponding to the djurleite phase and tetragonal chalcocite-Q phase, respectively. The difference in contrast is caused by the different Cu/Fe ratios in these two phases, with the details shown in Table SI.

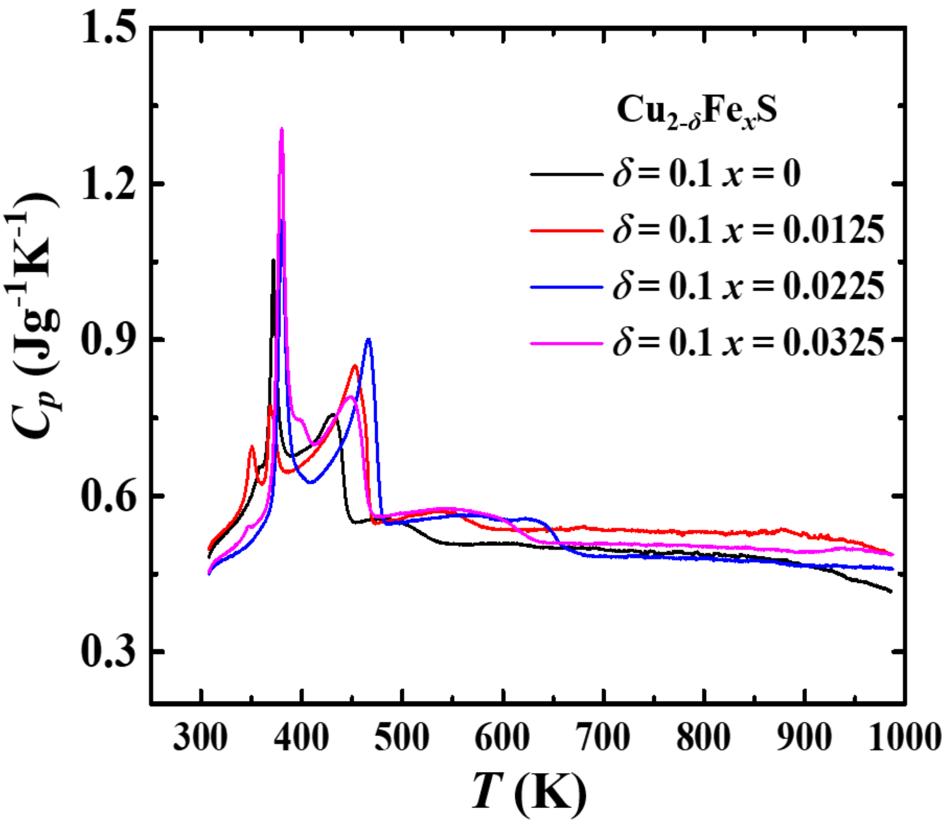

Figure S3 Temperature dependence of heat capacity for $Cu_{2-\delta}Fe_xS$ (δ = 0.1, x = 0, 0.0125, 0.0225, and 0.0325) at constant pressure (C_p).

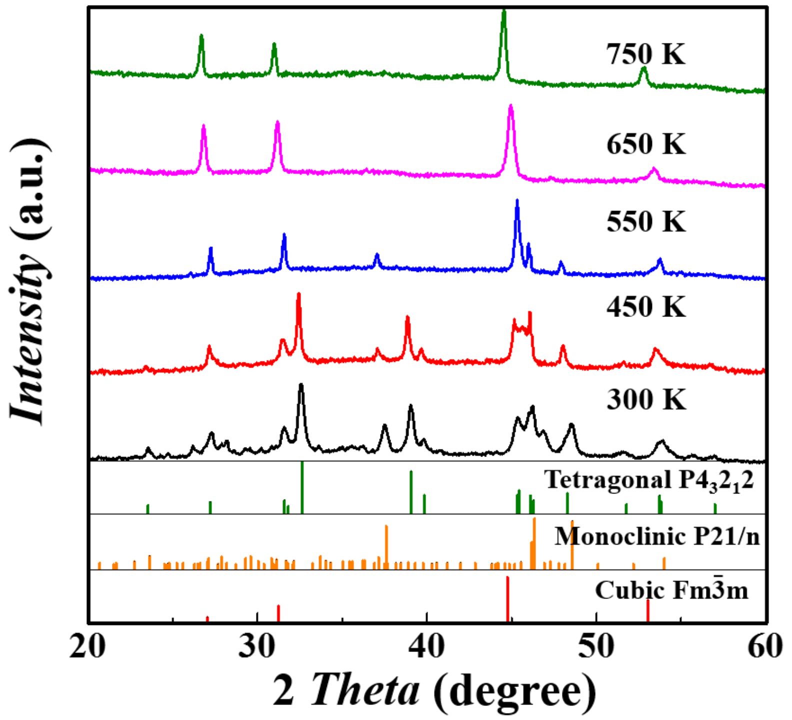

Figure S4 High temperature powder X-ray diffraction patterns of $Cu_{1.90}Fe_{0.0325}S$ measured from 300 K to 750 K.

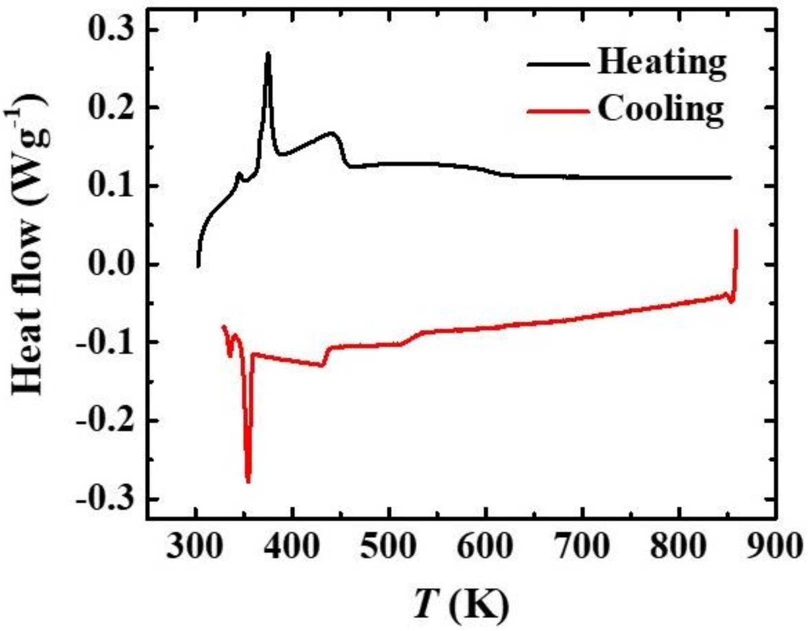

Figure S5 Heat flow curves of $Cu_{1.90}Fe_{0.0325}S$ during the heating and cooling process.

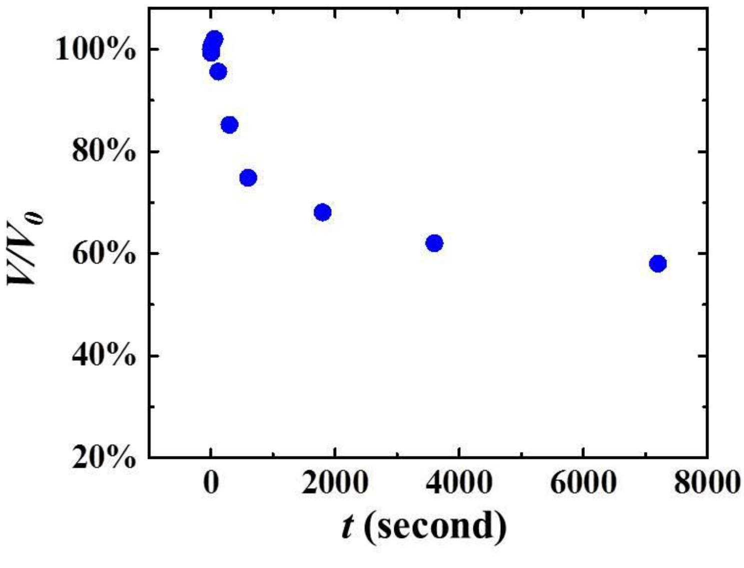

Figure S6 Relative voltage variation (V/V_0) of $Cu_{1.90}Fe_{0.0225}S$ as a function of current stress duration (t) under a temperature difference ΔT = 450 K. The test current density is J_t = 24 A cm^{-2}.

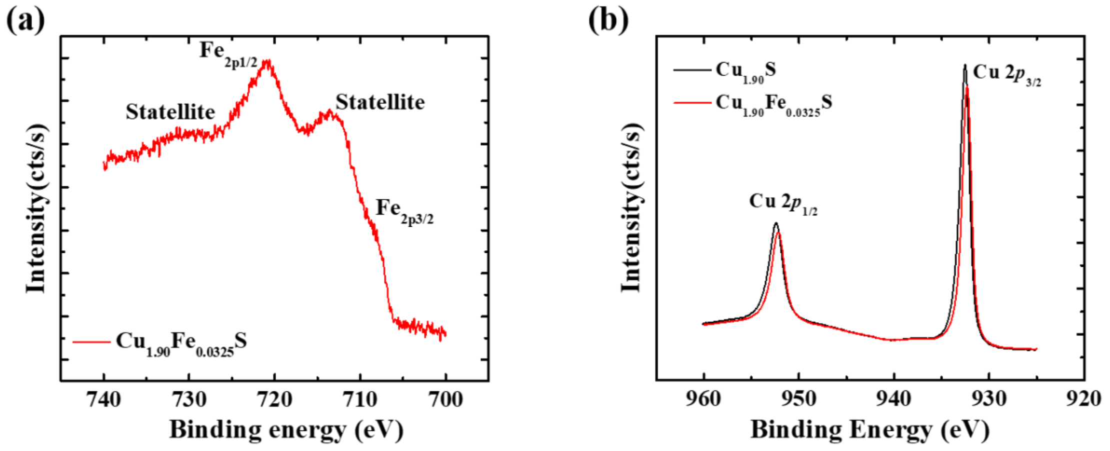

Figure S7 X-ray photoelectron spectroscopy spectra for $Cu_{1.90}Fe_{0.0325}S$. (a) Fe 2*p* and (b) Cu 2*p*. According to the analysis in Ref. S3-4, the valence states of Fe and Cu can be identified as +3 and +1, respectively [3,4]. The Cu 2p spectrum for the pristine Cu_2S taken from Ref. S5 is included in (b) for comparison [5].

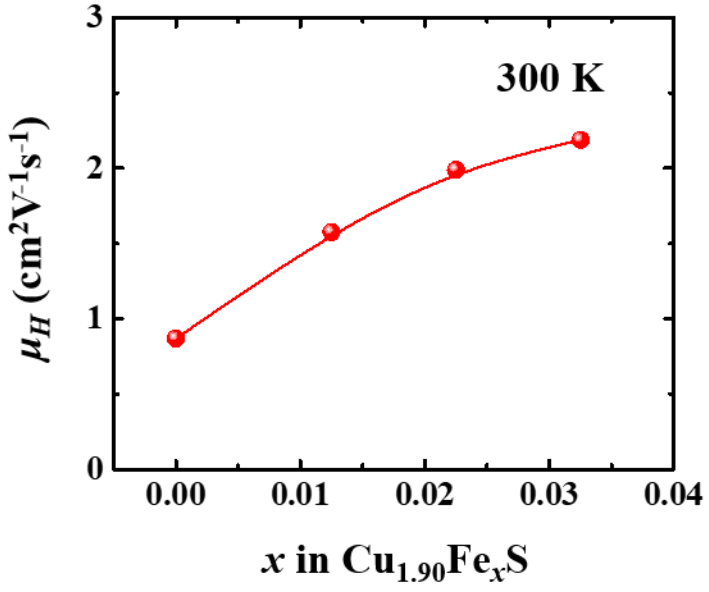

Figure S8 Carrier mobility (μ_H) as a function of the Fe doping content (x) at room temperature

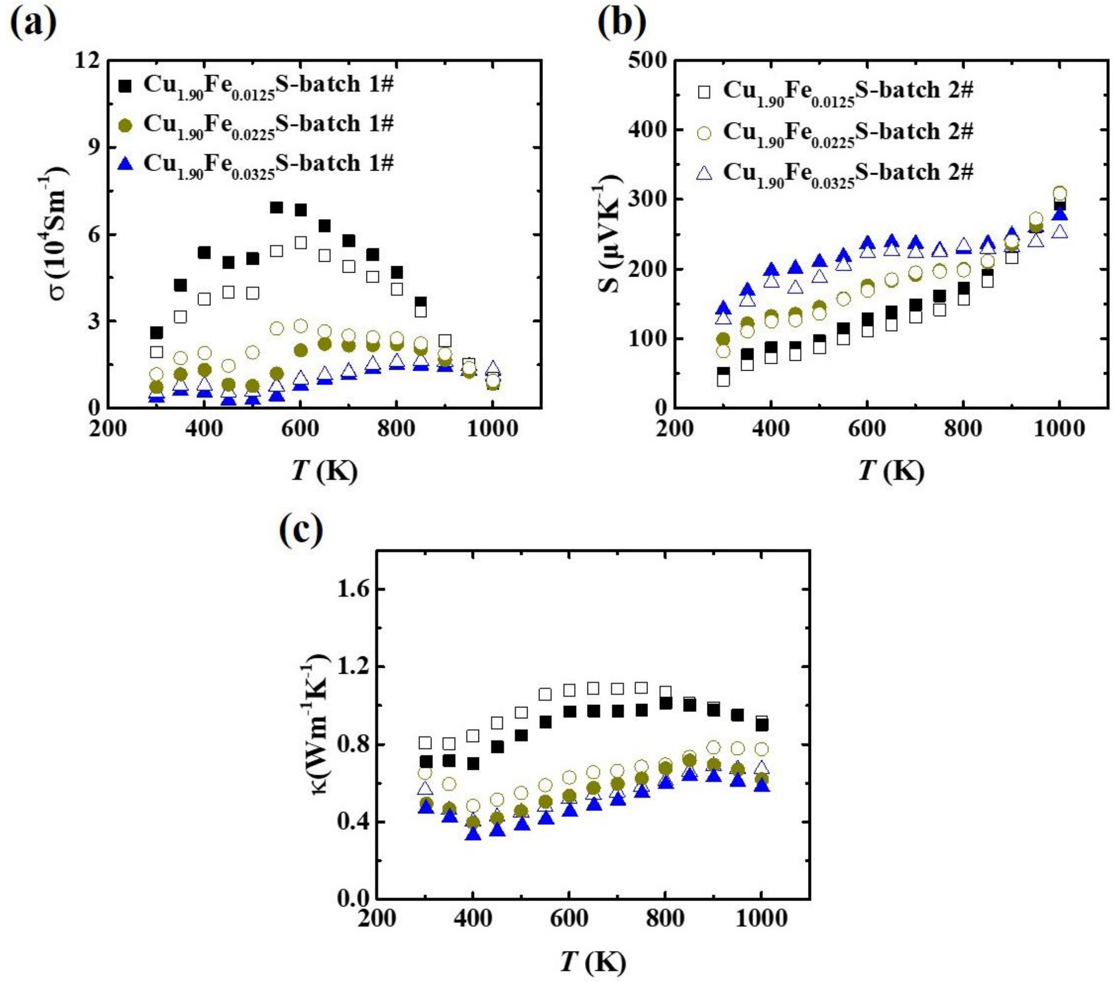

Figure S9 Temperature dependences of (a) electrical conductivity, (b) Seebeck coefficient, and (c) thermal conductivity for two batches of $Cu_{1.90}Fe_xS$ (x = 0, 0.0125, 0.0225, and 0.0325) samples.

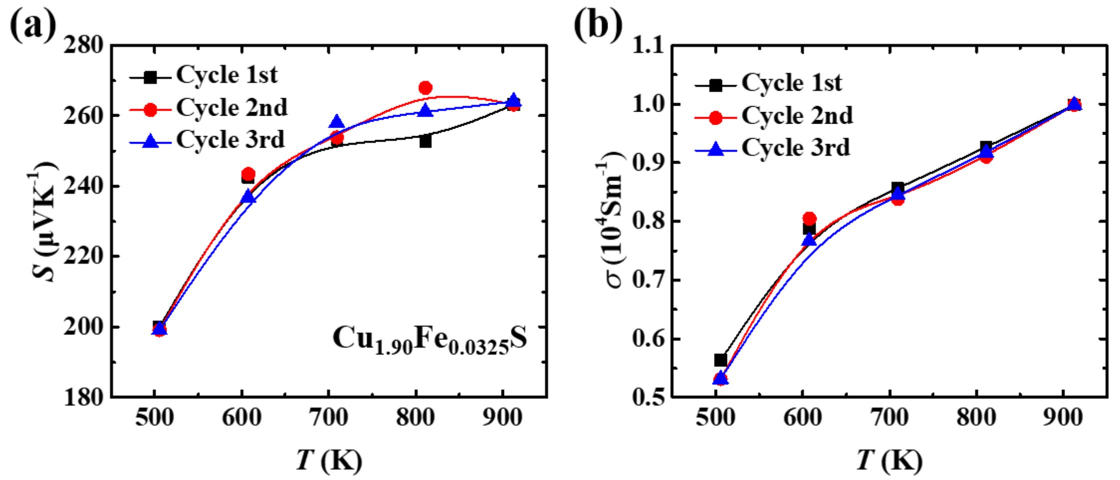

Figure S10 Repeatability test on electrical transport properties of $Cu_{1.90}Fe_{0.0325}S$ sample during cycling test.

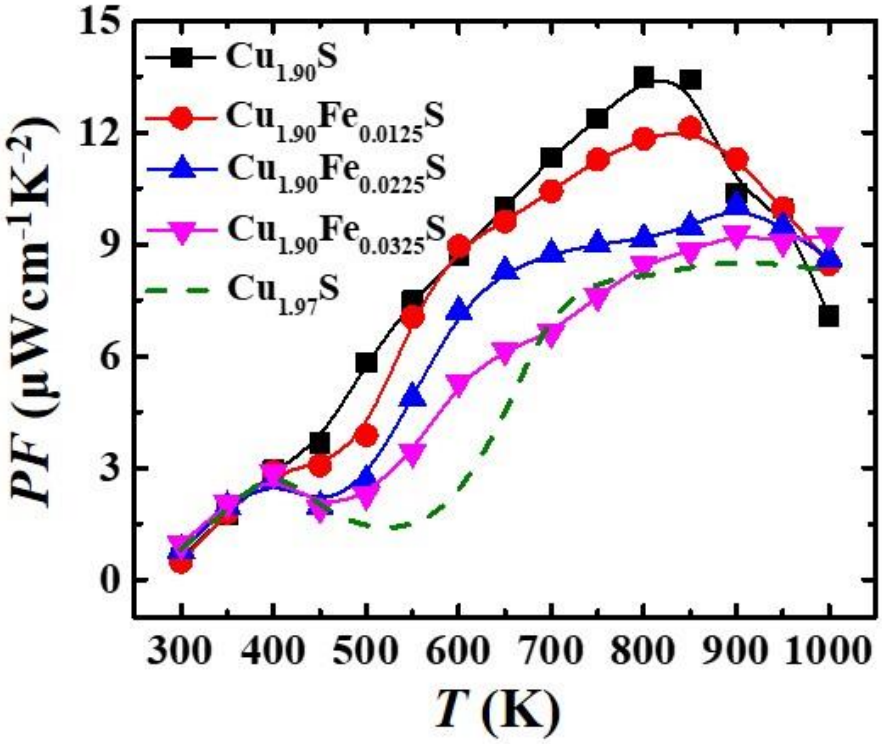

Figure S11 Temperature dependence of power factor (*PF*) for $Cu_{2-\delta}Fe_xS$ (δ = 0.1, x = 0, 0.0125, 0.0225, 0.0325). The data for $Cu_{1.97}S$ taken from Ref. S6 are also included for comparison [6].

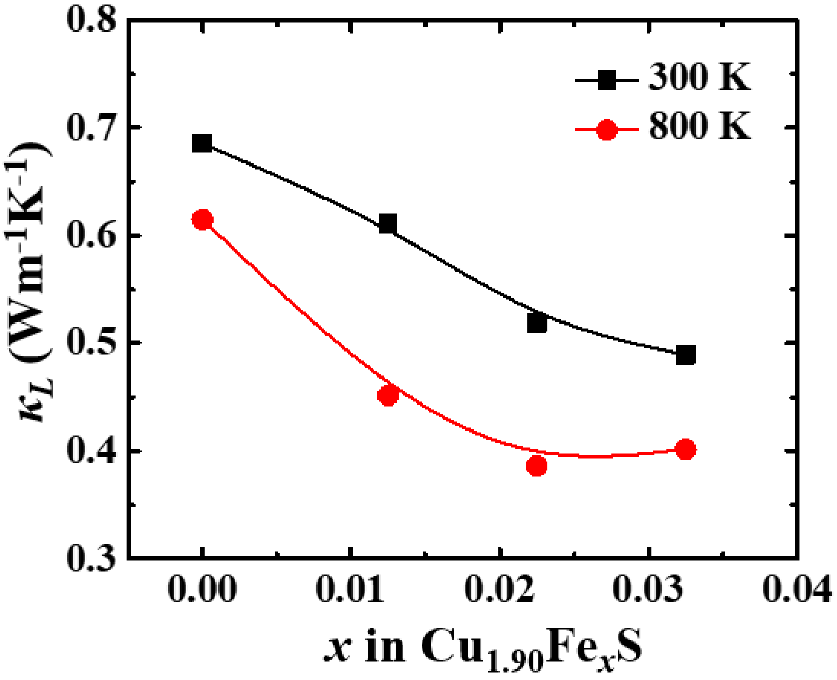

Figure S12 Lattice thermal conductivity (κ_L) as a function of Fe-doping content (x) for $Cu_{1.90}Fe_xS$ (x = 0, 0.0125, 0.0225, and 0.0325) at 300 K and 800 K.

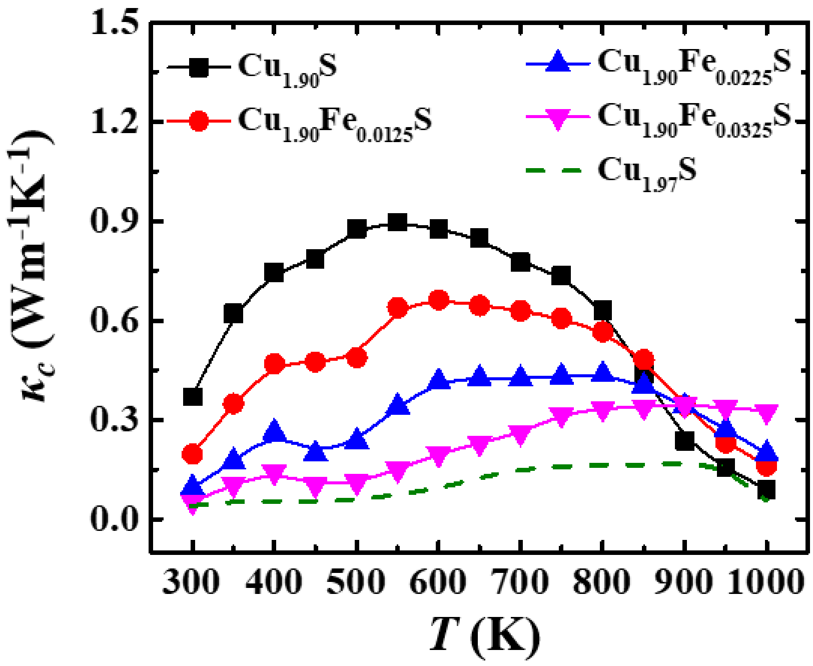

Figure S13 Temperature dependence of carrier thermal conductivity (κ_c) for $Cu_{2-\delta}Fe_xS$ (δ = 0.1, x = 0, 0.0125, 0.0225, 0.0325). The data for $Cu_{1.97}S$ taken from Ref. S6 are also included for comparison [6].

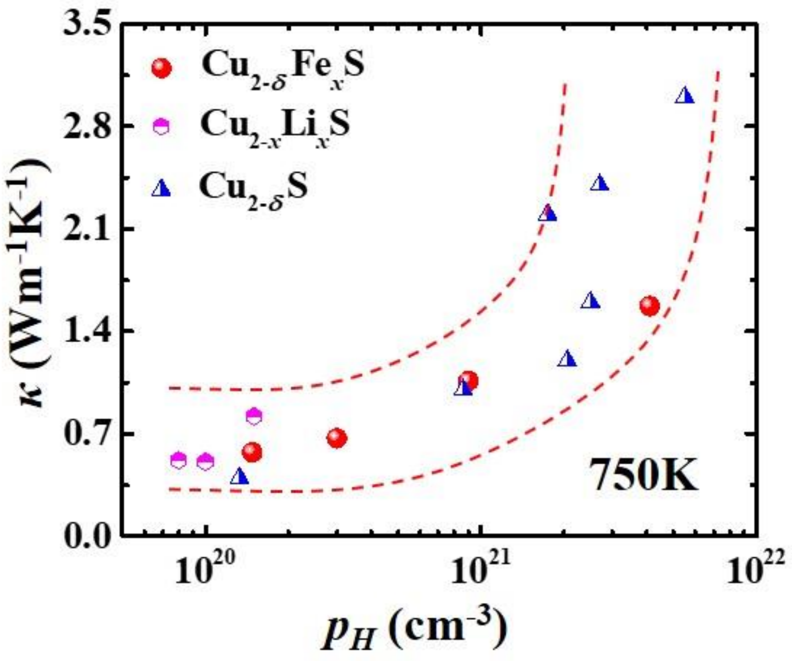

Figure S14 Hall carrier concentration (p_H) dependence of thermal conductivity (κ) for $Cu_{2-\delta}Fe_xS$ (δ = 0.1, x = 0, 0.0125, 0.0225, and 0.0325). The data for $Cu_{2-\delta}S$ (δ = 0, 0.03, 0.06, 0.08, and 0.1) and Li-doped Cu_2S are also included for comparison[7,8].

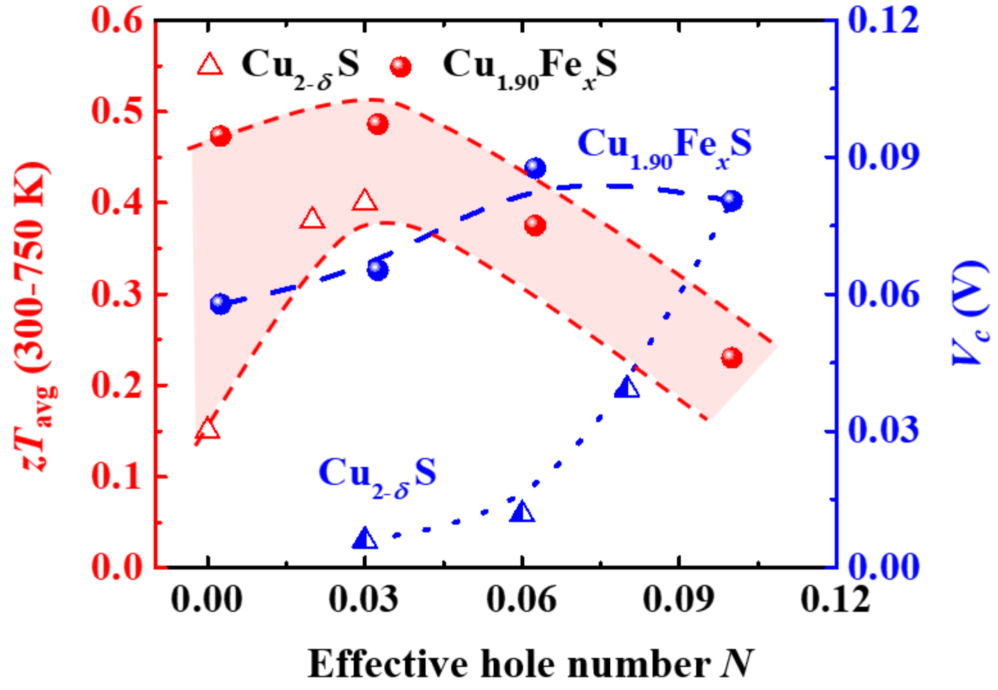

Figure S15 Average TE figure of merit (zT_{avg}) between 300-750 K and critical voltage (V_c) under the temperature difference of ΔT = 450 K as a function of the effective hole numbers (N) for $Cu_{2-\delta}S$ (δ = 0, 0.03, 0.06, 0.08, and 0.1) and $Cu_{1.90}Fe_xS$ (x = 0, 0.0125, 0.0225, and 0.0325). $N = \delta$ for $Cu_{2-\delta}S$ and $N = \delta - 3x$ for $Cu_{2-\delta}Fe_xS$.

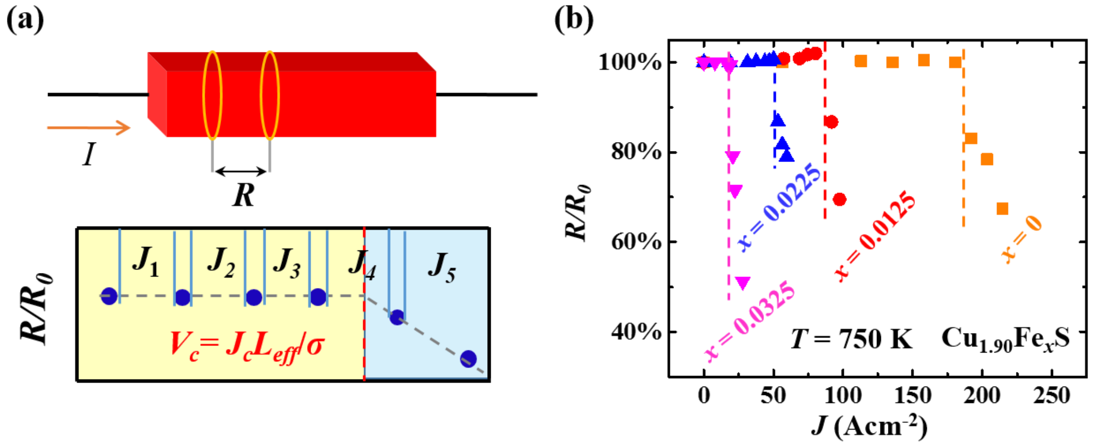

Figure S16 (a) Schematic of the critical voltage (V_c) measurement in the isothermal case. (b) Current density (J) dependence of relative electrical resistance variation (R/R_0) for $Cu_{2-\delta}Fe_xS$ (δ = 0.1, x = 0, 0.0125, 0.0225, and 0.0325) in the isothermal case with a constant temperature of 750 K. The dotted lines represent the critical condition when the Cu deposition occurs.

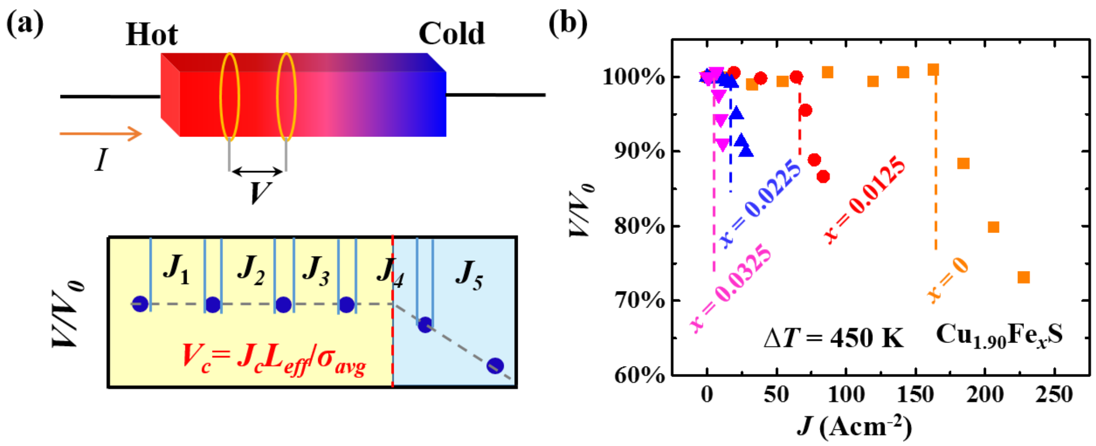

Figure S17 (a) Schematic of the critical voltage (V_c) measurement in the non-isothermal case. (b) Current density (J) dependence of relative voltage variation (V/V_0) for $Cu_{2-\delta}Fe_xS$ (δ = 0.1, x = 0, 0.0125, 0.0225, and 0.0325) in the non-isothermal case with the temperature difference of 450 K and hot side temperature of 750 K. The dotted lines represent the critical condition when the Cu deposition occurs.

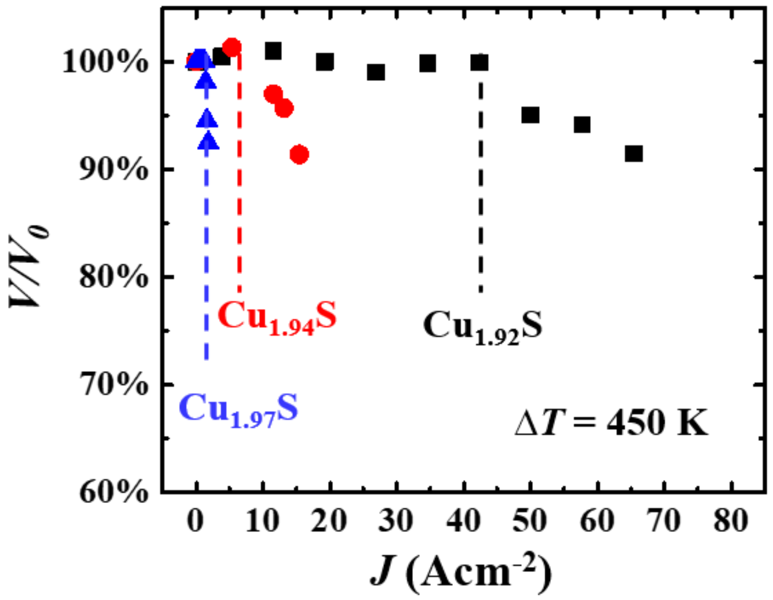

Figure S18 Current density dependence of relative voltage variation (V/V_0) for $Cu_{2-\delta}S$ (δ = 0.03, 0.06, and 0.08) in the isothermal case with a temperature difference ΔT = 450 K. The dotted lines represent the critical condition when the Cu deposition occurs.

Table. SI Actual Cu and Fe contents in the $Cu_{1.90}Fe_{0.0325}S$ determined by the EDS. The points are taken from the bright area and the gray area in Figure S2, respectively.

	**Point**	**Cu (%)**	**Fe (%)**	**Actual Cu/Fe ratio (average)**	**Nominal Cu/Fe ratio**
**Djurleite phase Contrast 1# (gray area)**	1	63.03	1.28	140/1	58/1
	2	63.12	1.20		
	3	63.36	0.90		
	4	63.39	1.01		
	5	63.14	1.09		
	6	63.31	1.18		
	7	63.21	1.05		
	8	63.01	1.54		
	9	63.70	1.06		
	10	62.89	1.40		
	11	63.15	1.25		
	12	63.86	0.89		
**Chalcocite-Q phase Contrast 2# (bright area)**	1	64.28	0.43	55/1	
	2	64.15	0.47		
	3	64.67	0.45		
	4	63.94	0.50		

Table SII Average electrical conductivity (σ_{avg}) among superionic phase (370-750 K) for $Cu_{2-\delta}S$ (δ = 0.03, 0.06, and 0.08).

**Composition**	**$Cu_{1.92}S$**	**$Cu_{1.94}S$**	**$Cu_{1.97}S$**
**σ_{avg} ($10^4 Sm^{-1}$)**	6.23	3.63	1.45

Table SⅢ Electrical conductivity (σ) at 750 K and average electrical conductivity (σ_{avg}) among superionic phase (370-750 K) for $Cu_{2-\delta}Fe_xS$ (δ = 0.1, x = 0, 0.0125, 0.0225, and 0.0325). The L_{eff} is 5 mm for the measured samples.

**Composition**	**x = 0**	**x = 0.0125**	**x = 0.0225**	**x = 0.0325**
**σ_{750K} (10^4 Sm^{-1})**	8.1	3.94	2.14	1.34
**σ_{avg} (10^4 Sm^{-1})**	10.8	3.86	1.48	0.655

References

[1] P. F. Qiu, M. T. Agne, Y. Y. Liu, Y. Q. Zhu, H. Y. Chen, T. Mao, J. Yang, W. Q Zhang, S. M. Haile, W. G. Zeier, J. Janek, C. Uher, X. Shi, L. D. Chen, G. J. Snyder, Nat Commun. **2018**, *9*, 2910.

[2] Goldsmid, H. J. Introduction to thermoelectricity; Springer: Berlin, **2010**

[3] H. Hu, Z. P. Liu, B. J. Yang, X. Y. Chen, Y. T. Qian, *J. Cryst. Growth*. **2005**, *284*, 226.
[4] T. Yamashita, P. Hayes, *Appl. Surf. Sci*. **2008**, *254*, 2441.
[5] S. S. Mali, P. S. Patil, C. K. Hong, *ACS Appl. Mater. Interfaces* **2014**, *6*, 1688
[6] Y. He, T. Day, T. S. Zhang, H. L. Liu, X. Shi, L. D. Chen, G. J. Snyder, Adv. Mater. **2014**, *26*, 3974
[7] P. F. Qiu, Y. Q. Zhu, Y. T. Qin, X. Shi, L. D. Chen, *APL Materials*. **2016**, *4*, 104805.
[8] M. J. Guan, P. F. Qiu, Q. F. Song, J. Yang, D. D. Ren, X. Shi, L. D. Chen, Rare Metals, **2018**, *37*, 282.
